# Transmission of F12-related hereditary angioedema through a sperm donor

**DOI:** 10.3389/fimmu.2026.1799395

**Published:** 2026-03-11

**Authors:** Lluís Marquès, Laura Batlle-Masó, Eva Alcoceba, Silvia Lara, Aina Aguiló-Cucurull, Johana Gil-Serrano, Mar Guilarte, Roger Colobran

**Affiliations:** 1 Allergy Department, Hospital Universitari Arnau de Vilanova, Institut de Recerca Biomèdica de Lleida (IRBLlleida), Lleida, Spain; 2 Translational Immunology Group, Vall d’Hebron Research Institute (VHIR), Barcelona, Spain; 3 Immunology Division, Hospital Universitari Vall d’Hebron (HUVH), Barcelona, Spain; 4 Allergy Department, Hospital Universitari de Santa Maria, Institut de Recerca Biomèdica de Lleida (IRBLlleida), Lleida, Spain; 5 Department of Allergy, Hospital Universitari Vall d’Hebron (HUVH), Barcelona, Spain; 6 Allergy Research Unit, Vall d’Hebron Research Institute (VHIR), Barcelona, Spain; 7 Department of Medicine, Autonomous University of Barcelona (UAB), Bellaterra, Spain; 8 Department of Clinical and Molecular Genetics, Hospital Universitari Vall d’Hebron (HUVH), Barcelona, Spain; 9 Department of Cell Biology, Physiology and Immunology, Autonomous University of Barcelona (UAB), Bellaterra, Spain

**Keywords:** F12 gene, HAE-FXII, hereditary angioedema, sperm donor, T328K variant

## Abstract

Hereditary angioedema due to *F12* pathogenic variants (HAE-FXII) is a rare autosomal dominant disorder characterized by recurrent episodes of angioedema mediated by bradykinin, incomplete penetrance, and marked sex-dependent clinical expression. Most affected individuals carry the recurrent *F12* c.983C>A/p.Thr328Lys (T328K) founder variant. Here, we report the first documented transmission of HAE-FXII through sperm donation. The index case was an 18-year-old woman with recurrent estrogen-dependent angioedema, normal C1-inhibitor levels and function, and poor response to conventional antiallergic therapy. Molecular analysis identified the heterozygous *F12* T328K variant. Familial investigation revealed the same variant in her asymptomatic twin brother, while their mother tested negative, strongly suggesting paternal transmission. The fertility clinic was contacted, and genetic testing confirmed that the sperm donor was a heterozygous carrier of the *F12* T328K variant. Women who had conceived using this donor’s sperm were subsequently notified. Through this process, a second unrelated family was identified, in which a young woman was also found to carry the heterozygous *F12* T328K variant and is currently asymptomatic under specialist follow-up. This study provides the first direct evidence of transmission of HAE-FXII through assisted reproductive technologies. Our findings highlight how autosomal dominant disorders with incomplete and sex-dependent penetrance can remain clinically silent in donors and be unknowingly transmitted to multiple offspring. These observations have important implications for genetic counseling, donor notification strategies, and donor screening policies, and support consideration of targeted screening for recurrent pathogenic variants such as *F12* T328K in regions where HAE-FXII is more prevalent.

## 1 Introduction

Hereditary angioedema (HAE) is a rare genetic disorder characterized by recurrent, self-limiting episodes of subcutaneous and submucosal edema affecting the skin, gastrointestinal tract, and upper airways. Clinically, patients may present with swelling of the extremities, face, or genitals, severe abdominal pain due to intestinal wall edema, and potentially life-threatening laryngeal edema. Unlike allergic angioedema, HAE attacks are not associated with urticaria or pruritus and do not respond to antihistamines, corticosteroids, or epinephrine (1). The estimated prevalence of HAE is approximately 1:50,000; however, this estimate likely varies among countries and largely reflects differences in diagnostic awareness and availability of appropriate diagnostic tools (2). Typically, the underlying pathophysiological mechanism in HAE is excessive generation of bradykinin, a potent vasoactive peptide that increases vascular permeability and leads to edema formation (3).

The most frequent form of HAE is caused by heterozygous pathogenic variants in the *SERPING1* gene, encoding the C1-Inhibitor (C1INH), a multifunctional plasma serine protease inhibitor involved in the regulatory network of complement, contact, coagulation, and fibrinolytic systems (4). In HAE due to C1 inhibitor deficiency (HAE-C1INH), reduced levels or impaired function of C1INH result in inadequate control of the contact system, with persistent activation of factor XII and plasma kallikrein, culminating in excessive bradykinin production and angioedema formation. To date, more than 800 different pathogenic variants in *SERPING1* have been described, distributed throughout the entire gene, including missense, nonsense, splice-site variants, small insertions/deletions, and large rearrangements (5). HAE-C1INH accounts for approximately 85-90% of all HAE cases, with pathogenic variants classically classified as causing type I HAE, characterized by a quantitative deficiency of C1INH, or type II HAE, characterized by qualitative dysfunction despite normal or elevated antigenic C1INH levels.

In addition to HAE-C1INH, a subset of patients presents with a clinical phenotype consistent with HAE but with normal levels and function of C1INH. This group is currently referred to as hereditary angioedema with normal C1 inhibitor (HAE-nC1INH). Over the past two decades, significant progress has been made in elucidating the genetic basis of these forms, leading to the identification of eight different genes associated with HAE-nC1INH: *F12* (HAE-FXII) (6), *PLG* (HAE-PLG) (7), *ANGPT1* (HAE-ANGPT1) (8), *KNG1* (HAE-KNG1) (9), *MYOF* (HAE-MYOF) (10), *HS3ST6* (HAE-HS3ST6) (11), *DAB2IP* (HAE-DAB2IP) (12) and *CPN1* (HAE-CPN1) (13). All these genes cause HAE with autosomal dominant inheritance, except for *CPN1*, which has been associated with an autosomal recessive form of the disease. However, despite the increasing number of genes identified in recent years, a substantial proportion of patients with HAE and normal C1 inhibitor do not carry pathogenic variants in any of these genes. These cases are therefore classified as hereditary angioedema of unknown genetic origin (HAE-UNK) (14).

**Figure 1:**
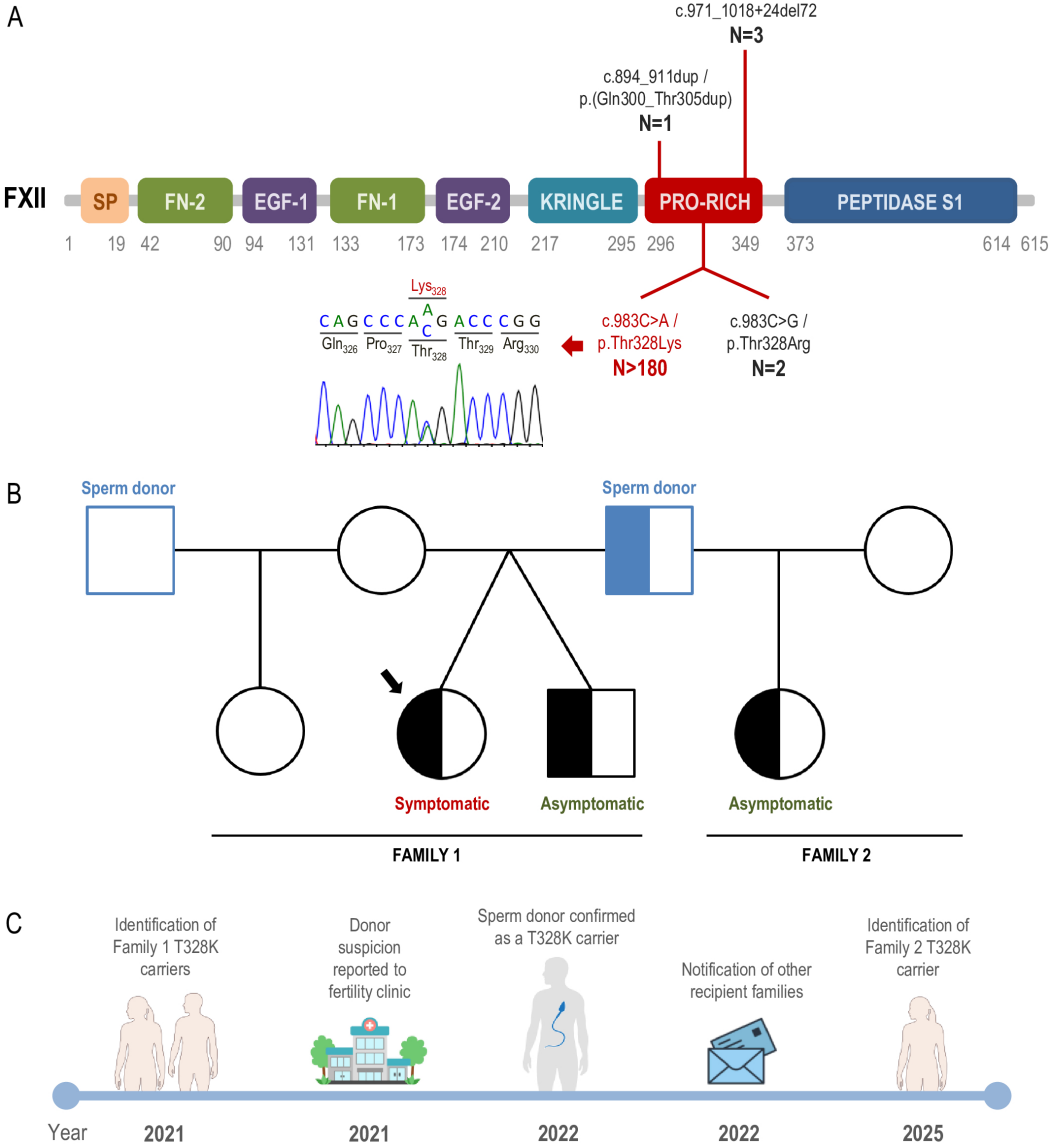
HAE-FXII transmitted through a sperm donor. **(A)** Schematic representation of the FXII protein showing the localization of the four pathogenic variants described in HAE-FXII. For each variant, the number (N) of reported families is indicated. The amino acid boundaries of the different FXII protein domains are shown. A representative Sanger sequencing chromatogram illustrating the *F12* c.983C>A/p.Thr328Lys (T328K) variant is also displayed. **(B)** Pedigrees of the two families reported in this study. Sperm donor symbols are depicted in blue. Half-filled symbols indicate heterozygous carrier status for the *F12* T328K variant. **(C)** Schematic timeline summarizing the key events of the present study. All images used to illustrate the timeline are license-free and were obtained from the NIAID NIH BioArt Source (https://bioart.niaid.nih.gov) and Vecteezy (https://www.vecteezy.com/).

Pathogenic variants in the *F12* gene are the second most common cause of hereditary angioedema, after *SERPING1*. The *F12* gene encodes coagulation factor XII (FXII), a key component of the contact activation system. Remarkably, only four different disease-causing variants have been reported so far in *F12*, all of them localized in exon 9 of the gene (6, 15, 16) (**Figure 1A**). More than 98% of affected individuals carry the same recurrent missense variant, c.983C>A/p.Thr328Lys (T328K), strongly suggesting a founder effect (17, 18). This striking mutational homogeneity contrasts sharply with the extensive allelic heterogeneity observed in the *SERPING1* gene in HAE-C1INH.

The pathogenic mechanism underlying HAE-FXII is distinct from that of HAE-C1INH. The T328K variant introduces a novel cleavage site for plasmin and thrombin within the FXII protein, leading to increased susceptibility to proteolytic activation in fluid phase (19, 20). As a result, mutant FXII can be activated independently of the classical contact pathway, promoting excessive generation of kallikrein and subsequent overproduction of bradykinin. This gain-of-function mechanism explains why patients exhibit normal C1INH levels and activity while still developing bradykinin-mediated angioedema. Importantly, this pathway is particularly sensitive to estrogen modulation, which has major clinical implications.

A key feature of HAE-FXII is its incomplete penetrance. This incomplete penetrance is strongly sex-dependent. In male carriers of the T328K variant, disease penetrance is very low, estimated to be below 10%, whereas in female carriers penetrance is significantly higher, reaching approximately 60-80%, although still incomplete (17). The marked sex bias is largely explained by hormonal factors. Estrogens can upregulate components of the contact system, which may enhance bradykinin generation and thereby increase susceptibility to angioedema. Notably, expression of the *F12* gene is positively regulated by estrogens due to the presence of estrogen-responsive elements in its promoter region (21). Clinically, disease onset or worsening in women is frequently associated with estrogen exposure, including puberty, pregnancy, oral contraceptive use, or hormone replacement therapy. Conversely, symptoms may improve after menopause or upon withdrawal of exogenous estrogens.

The combination of incomplete penetrance and marked sex dependency has important implications for inheritance patterns and genetic counseling. In particular, the low penetrance in men may allow the pathogenic variant to be silently transmitted across generations without overt clinical manifestations. Asymptomatic male carriers may therefore unknowingly pass the mutation to multiple descendants, in whom the disease may only become apparent in female carriers or under specific hormonal conditions. This phenomenon can obscure family history and delay diagnosis, especially in non-classical transmission settings.

Here we report, for the first time, two unrelated families in which the recurrent *F12* T328K variant, was transmitted through the same sperm donor.

## 2 Methods

### 2.1 Subjects of the study

The reported families were attended at the Allergy Division of Hospital Universitari de Santa Maria (Lleida, Spain) (Family 1) and the Allergy Division of Hospital Universitari Vall d’Hebron (Barcelona, Spain) (Family 2). Written informed consent for the studies reported here and for publication of the article was obtained from both families, according to the procedures of the Clinical Research Ethics Committee of the Vall d’Hebron University Hospital [code: PR(AG)202/2021].

### 2.2 Laboratory tests

Serum levels of C3, C4, and C1INH were determined by turbidimetry (Optilite, Binding Site, UK). C1INH functional activity was determined by ELISA (QuidelOrtho, USA).

### 2.3 *F12* molecular screening

Genomic DNA was extracted from EDTA-containing whole blood using a QIAamp DNA Mini Kit (Qiagen, Germany) according to the manufacturer’s instructions. The exon 9 of *F12* gene was amplified by PCR (primers and PCR conditions are available upon request). Purified PCR products were sequenced by Sanger method using an Applied Biosystems 3500 Genetic Analyzer (Thermo Fisher Scientific, USA).

## 3 Results

### 3.1 Suspected transmission of the *F12* T328K mutation through sperm donation in a family with two carrier siblings

We report a family (Family 1) with no previous history of angioedema, in which the index patient was a female who presented at 18 years of age with an isolated episode of facial angioedema without identifiable triggers. One year later, she experienced a second episode of lip edema, again without identifiable triggers, requiring emergency care. Five days later, she developed unilateral foot edema, followed by laryngeal tightness with mild dyspnea and sublingual edema. Despite treatment with antihistamines and corticosteroids, symptoms did not improve and progressed to involve the tonsillar pillars. She subsequently received intramuscular epinephrine and was admitted to emergency department, with poor clinical response.

One week later, she was readmitted with severe facial edema, showing only slow clinical improvement. Prophylactic treatment with cetirizine 20 mg daily was initiated. No triggering factors were identified, and she denied the use of nonsteroidal anti-inflammatory drugs. One week thereafter, she developed mild facial edema associated with palatal edema, throat tightness, and dysphonia, again with poor response to antihistamines and corticosteroids, leading to hospital readmission. At discharge, tranexamic acid 2 g daily was added to baseline cetirizine and a prolonged tapering course of oral corticosteroids. In the episodes for which duration was recorded, symptoms persisted beyond 48 hours.

The patient denied abdominal pain, trauma-induced angioedema, or urticaria. Her medical history included egg allergy in remission and mild seasonal rhinoconjunctivitis. There was no known family history of angioedema. Laboratory evaluation showed a normal complete blood count and a comprehensive metabolic panel without alterations. Complement studies were normal, including C4 levels (23 mg/dL, normal range: 13–40 mg/dL) and C1 inhibitor functional activity (93%), effectively ruling out HAE-C1INH.

During both periods of angioedema, the patient was taking estrogen-containing oral contraceptives, which she had initiated several weeks before her first episode. Oral contraceptives were discontinued during the COVID-19 general lockdown, with no further episodes. One year later, she resumed estrogen-containing contraceptives, and angioedema recurred within a few days. Given this temporal association, oral contraceptives were discontinued again, resulting in complete symptom resolution and gradual withdrawal of prophylactic cetirizine and tranexamic acid.

The clear estrogen dependence of angioedema episodes strongly suggested a bradykinin-mediated mechanism and prompted molecular analysis of the *F12* gene. This analysis identified the heterozygous c.983C>A/p.Thr328Lys (T328K) variant, the most frequently reported pathogenic variant associated with HAE-FXII. A subsequent familial segregation study was therefore undertaken. The patient had two siblings, an older sister and a twin brother, all conceived via *in vitro* fertilization using sperm from two different anonymous donors (**Figure 1B**, Family 1). During the patient’s gestation, three embryos fertilized with sperm from a single anonymous donor were implanted, two of which developed successfully. Genetic testing of the patient’s mother did not identify the variant, effectively excluding maternal transmission. The twin brother was found to carry the same heterozygous *F12* T328K variant. At the time of diagnosis, he was asymptomatic and reported only a single episode of mild lip edema without any evident trigger. He denied any history of abdominal pain or trauma-induced angioedema.

During a four-year follow-up period, the index patient experienced several episodes of mild facial or peripheral angioedema, some of which were successfully treated with subcutaneous icatibant (30 mg). In the episodes for which response time was documented (n=4), clinical improvement was noted within the first 30 minutes after icatibant administration. She has tolerated progestogen-only oral contraception (drospirenone) without recurrence of estrogen-associated symptoms.

### 3.2 Confirmation of *F12* T328K mutation transmission through a sperm donor

Given that both the index patient and her twin brother carried the *F12* T328K variant while the mother tested negative, paternal transmission through the sperm donor was strongly suspected. This interpretation is supported by several considerations. First, the T328K variant is a well-established recurrent pathogenic variant causing HAE-FXII and, to date, has only been reported as an inherited variant, with no confirmed *de novo* cases. Second, although a *de novo* event cannot be entirely excluded, the presence of the same variant in two siblings makes this possibility exceedingly unlikely. Finally, maternal gonadal mosaicism represents an alternative theoretical explanation but is considered far less probable than transmission through the sperm donor.

Accordingly, the fertility clinic was contacted and informed of the situation, and genetic testing of the sperm donor was requested. The fertility clinic then liaised with the sperm bank, and genetic analysis of the donor confirmed that he was a heterozygous carrier of the *F12* T328K variant (**Figure 1B**). Sperm donor anonymity was preserved throughout the study in accordance with Spanish regulations on assisted reproductive technologies (Law 14/2006 on Assisted Human Reproduction Techniques).

### 3.3 Identification of an additional *F12* T328K carrier from the same sperm donor

Following confirmation that the sperm donor was a heterozygous carrier of the *F12* T328K variant, the fertility clinic initiated a notification process addressed to all women who had previously received sperm from this donor and had biological offspring. The notification letter informed recipients of the identification of the *F12* T328K variant in the donor and provided an overview of the clinical condition associated with this variant (HAE-FXII). The letter specified the autosomal dominant mode of inheritance of the disease and highlighted the resulting 50% probability of variant transmission to offspring. In addition, it described the sex-dependent and incomplete penetrance of HAE-FXII, emphasizing the low penetrance observed in male carriers, the higher -although still incomplete- penetrance in females, and the strong influence of hormonal factors on disease expression. Finally, the letter provided information on how to proceed should the families wish to pursue genetic testing of their offspring to determine carrier status for the *F12* T328K variant. For confidentiality reasons, we do not have access to information regarding the total number of women who received sperm from this donor or the number of offspring conceived.

Several months later, in mid-2025, a woman attended the Allergy Department of Hospital Universitari Vall d’Hebron (Barcelona, Catalonia, Spain) after receiving a notification letter from her fertility clinic indicating that the sperm donor used for conception was a carrier of the *F12* T328K variant. As immediately suspected, the fertility clinic involved was the same as that attending the family described above, and the sperm donor was confirmed to be identical. The woman had a daughter conceived with sperm from this donor and, following receipt of the notification, requested genetic testing. Molecular analysis demonstrated that the daughter was a heterozygous carrier of the *F12* T328K variant (**Figure 1B**, Family 2). At the time of evaluation, the patient (21-year-old) was asymptomatic and is currently under clinical follow-up at the Allergy Department.

## 4 Discussion

In this study, we report an unusual case of HAE-FXII transmitted through sperm donation, resulting in the identification of other heterozygous carriers from the same anonymous donor. To our knowledge, this represents the first documented report of transmission of the recurrent *F12* T328K variant through assisted reproductive technologies, with molecular confirmation of donor carrier status and subsequent identification of additional affected offspring. These findings have important implications for the diagnosis of HAE-FXII, genetic counseling in assisted reproduction settings, and donor screening policies.

The initial presentation of the index patient illustrates several hallmark features of HAE-FXII. Despite the absence of family history, she developed recurrent episodes of angioedema with poor response to antihistamines, corticosteroids, and epinephrine, normal complement parameters, and a strong temporal relationship with estrogen exposure. These characteristics are highly suggestive of bradykinin-mediated angioedema and underscore the importance of considering HAE-FXII in young women with estrogen-dependent angioedema and normal C1 inhibitor function. In such cases, molecular analysis of *F12* is critical to establish the diagnosis and avoid misclassification as idiopathic or allergic angioedema (22).

The identification of the same *F12* T328K variant in the asymptomatic twin brother highlights another key feature of HAE-FXII: its markedly incomplete and sex-dependent penetrance. Male carriers frequently remain asymptomatic or experience only mild or situational symptoms, which may easily go unrecognized. In the present family, the twin brother reported only a single episode of mild lip edema. This low penetrance in males is well documented and allows pathogenic *F12* variants to be transmitted silently across generations (23). This phenomenon acquires particular relevance in the context of sperm donation. Although no clinical information about the donor was available due to confidentiality constraints, it is plausible that he had no history of angioedema and therefore would not have met any exclusion criteria during routine donor selection. Nevertheless, he was a heterozygous carrier of the most frequent pathogenic variant (T328K) associated with HAE-FXII. The geographic distribution of HAE-FXII due to the T328K variant is highly non-uniform. The highest concentration of reported cases has been described in the Iberian Peninsula, particularly in Spain and Portugal, followed by France and Brazil (17). Additional cases have been reported in other South American countries, several European regions, and North Africa, especially Morocco. This distinctive distribution supports the hypothesis of a founder mutation that has spread through specific populations via historical migration patterns. Given that the fertility clinic and both families described in this study are located in Catalonia, it is plausible that the donor originated from the Iberian Peninsula.

The confirmation of donor carrier status, followed by the identification of a second heterozygous carrier from an unrelated family conceived using sperm from the same donor, illustrates the potential for assisted reproductive technologies to amplify the dissemination of pathogenic variants (24). Although the present study represents the first reported case of HAE transmitted through sperm donation, previous reports have documented the transmission of other genetic diseases through gamete donation (**Table 1**). In most reported cases, the transmitted disorders followed an autosomal dominant or X-linked recessive pattern of inheritance. For autosomal dominant conditions, donors were typically asymptomatic or only very mildly affected, with clinical manifestations not detected during standard donor screening, despite carrying pathogenic germline variants. In other instances, transmission was attributed to gonadal mosaicism in the donor. Reported cases of X-linked recessive disorders predominantly involved oocyte donation from asymptomatic carrier donors (**Table 1**).

**Table 1:** Reported cases of genetic disease transmission through gamete donation (1990–2026).

Genetic disease	Inheritance	Gene	Year of publication	Country	Donation type	Number of affected cases^a^	Key observations	Reference
Autosomal Dominant Cerebellar Ataxia (ADCA)	AD	Not specified	2002	Netherlands	Sperm	18 children in 13 women	◦ Donor disease discovered disease years after donation. ◦ Late-onset manifestation (post-puberty). ◦ Parents notified 3 years after insemination.	(27)
Fragile X Syndrome (premutation)	XL dominant	*FMR1*	2008	USA	Sperm	1 affected girl	◦ Donor with premutation transmitted to daughter. ◦ Asymptomatic male donor. ◦ First documented case of FXS transmission via sperm donation.	(28)
Hypertrophic Cardiomyopathy (HCM)	AD	*MYH7*	2009	USA	Sperm	9 (out of 24 children known to be offspring from the donor)	◦ Asymptomatic donor (23-year-old). ◦ 95 donations over 2 years. ◦ Novel MYH7 pathogenic variant (Arg169Gly) identified in the donor after offspring clinical diagnosis.	(29)
Neurofibromatosis Type 1 (NF1)	AD	*NF1*	2016	Denmark	Sperm	9 (out of 23 children known to be offspring from the donor)	◦ Germline mosaicism in donor. ◦ Mild/absent manifestations in the donor not detected by standard physical screening. ◦ Precedent to TP53 case in same country (see below).	(30)
Lowe Syndrome (Oculocerebrorenal)	XL recessive	*OCRL*	2019	Greece	Oocytes	3 (of 9 total births)	◦ *De novo* pathogenic variant (point mutation) in asymptomatic healthy donor with one healthy daughter. ◦ Donor had normal karyotype (does not detect point mutations). ◦ Oocyte donation to 5 couples.	(31)
Adrenoleukodystrophy (ALD)	XL recessive	*ABCD1*	2023	USA	Oocytes	3 children	◦ Asymptomatic carrier donor. ◦ Failure in X-linked disease screening. ◦ Severe neurological progression.	(32)
Li-Fraumeni Syndrome	AD	*TP53*	2025	Denmark → 14 EU countries	Sperm	At least 23 (out of 197 children known to be offspring from the donor).	◦ Germline mosaicism (20% sperm mutated). ◦ Donations from 2005 to 2022. ◦ 90% cancer risk by age 60. ◦ National limits vastly exceeded (38 families in Belgium alone *vs*. limit of 6). ◦ Major European scandal.	(33)
Hereditary Angioedema (HAE-FXII)	AD	*F12*	2026	Spain	Sperm	3 children in 2 families	◦ Donation >20 years ago. ◦ Asymptomatic male donor (<10% penetrance in men). ◦ Detected after symptoms in young woman upon starting oral contraceptives. ◦ Not included in screening protocols.	This study

a Number of individuals who inherited the pathogenic variant, independent of clinical phenotype.

Following the identification of two carriers in Family 1 and the strong suspicion of paternal transmission through the sperm donor, the fertility clinic was promptly contacted. The coordinated and timely response of the clinic, including notification of recipient mothers, represents an example of good clinical and ethical practice (**Figure 1C**). Although confidentiality constraints precluded direct access to information regarding the total number of offspring conceived from this donor, the notification strategy enabled the identification of at least one additional heterozygous carrier (Family 2), who was subsequently placed under specialist follow-up prior to symptom onset. This proactive approach is consistent with the principles of beneficence and harm prevention, while maintaining respect for donor anonymity and privacy (25).

These findings raise important questions regarding genetic screening practices in sperm donation programs. Current donor screening protocols primarily focus on infectious diseases and, in some settings, on a limited panel of recessive genetic conditions with relatively high carrier frequencies. In contrast, autosomal dominant disorders with incomplete penetrance -particularly those that may remain clinically silent in young adults- are generally not included in routine screening strategies (26). While universal genetic screening for all possible dominant conditions is neither practical nor feasible, our study illustrates that selected disorders may warrant specific consideration. HAE-FXII represents a paradigmatic example, given its potential for severe and life-threatening manifestations, its strong hormonal modulation with delayed or sex-dependent clinical expression, and the availability of effective targeted therapies. In this context, targeted screening for HAE-FXII, particularly for the recurrent *F12* T328K variant, in sperm donors from regions where the condition is more prevalent could be considered a reasonable and proportionate approach.

In conclusion, this study documents the transmission of the *F12* T328K pathogenic variant through sperm donation, resulting in multiple heterozygous carriers and highlighting the challenges posed by autosomal dominant disorders with incomplete, sex-dependent penetrance in assisted reproduction. As the use of assisted reproductive technologies continues to expand, careful evaluation of how to manage rare but clinically significant genetic conditions will be increasingly important to ensure optimal patient care and informed decision-making.

## Data Availability

The datasets generated and analyzed during the current study consist of patient-derived genetic data obtained for clinical diagnostic purposes. Due to ethical, legal, and privacy restrictions and in accordance with institutional review board approvals and informed consent, these data cannot be made publicly available. No novel genomic datasets were generated that are suitable for deposition in a public repository. Relevant information supporting the findings of this study is included within the article. Additional anonymized data may be made available from the corresponding author upon reasonable request and subject to institutional approval.
